# Internet based intervention (Emotion Regulation Individual Therapy for Adolescents) as add‐on to treatment as usual versus treatment as usual for non‐suicidal self‐injury in adolescent outpatients: The TEENS randomised feasibility trial

**DOI:** 10.1002/jcv2.12115

**Published:** 2022-12-03

**Authors:** Britt Morthorst, Markus Harboe Olsen, Janus Christian Jakobsen, Jane Lindschou, Christian Gluud, Michella Heinrichsen, Bo Møhl, Lotte Rubæk, Olivia Ojala, Clara Hellner, Johan Bjureberg, Anne Katrine Pagsberg

**Affiliations:** ^1^ Research Unit Child and Adolescent Mental Health Center Copenhagen University Hospital—Mental Health Services CPH Copenhagen Denmark; ^2^ Department of Clinical Medicine Faculty of Health University of Copenhagen Copenhagen Denmark; ^3^ Copenhagen Trial Unit Centre for Clinical Intervention Research The Capital Region Copenhagen University Hospital—Rigshospitalet Copenhagen Denmark; ^4^ Department of Neuroanaesthesiology Neuroscience Centre Copenhagen University Hospital—Rigshospitalet Copenhagen Denmark; ^5^ Department of Regional Health Research The Faculty of Health Sciences University of Southern Denmark Odense Denmark; ^6^ Department of Communication and Psychology Aalborg University Aalborg Denmark; ^7^ Team of Self‐Injury Child and Adolescent Mental Health Services Copenhagen Denmark; ^8^ Department of Clinical Neuroscience Centre for Psychiatry Research Karolinska Institutet, & Stockholm Health Care Services Region Stockholm Stockholm Sweden; ^9^ Department of Psychology Stanford University Stanford California USA

**Keywords:** emotion regulation individual therapy for adolescents, ERITA, feasibility trial, internet based intervention, non‐suicidal self‐injury, randomised

## Abstract

**Background:**

Non‐suicidal self‐injury (NSSI) is common in adolescents receiving psychiatric treatment and is a significant risk factor for suicidal behavior. There are few randomised clinical trials assessing interventions for NSSI in youth, and knowledge about internet‐delivered interventions is limited.

**Objective:**

We assessed the feasibility of Internet based Emotion Regulation Individual Therapy for Adolescents (ERITA) in psychiatric outpatients aged 13–17 years who engaged in NSSI.

**Method:**

A randomised clinical feasibility trial with a parallel group design. Non‐suicidal self‐injury engaging patients were recruited from Child and Adolescent Mental Health Outpatient Services in the Capital Region of Denmark from May to October 2020. ERITA was provided as add‐on to treatment as usual (TAU). ERITA is a therapist‐guided, internet‐based program of emotion regulation and skills training involving a parent. The control intervention was TAU. Feasibility outcomes were the proportion who completed follow‐up interviews at end of intervention; proportion of eligible patients who participated in the trial; proportion of participants completing ERITA. We further investigated relevant exploratory outcomes, including adverse risk‐related events.

**Results:**

We included 30 adolescent participants, 15 in each group (ERITA vs. Treatment as usual). 90% (95% CI, 72%–97%) of the participants completed post‐treatment interviews; 54% (95% CI, 40%–67%) of the eligible participants were included and randomised; and 87% (95% CI, 58%–98%) of the participants completed at least six out of 11 ERITA modules. We identified no difference for the primary exploratory clinical outcome of NSSI between the two groups.

**Conclusion:**

There are few randomised clinical trials assessing interventions for NSSI in youth, and knowledge about internet‐delivered interventions is limited. Based on our results we conclude that a large‐scale trial seems feasible and warranted.

## Graphical abstract

Internet based intervention for non‐suicidal self‐injury (ERITA) is tested in Child and Adolescent Mental Health Services in Denmark and found feasible warranting for further investigation in a large‐scale trial.

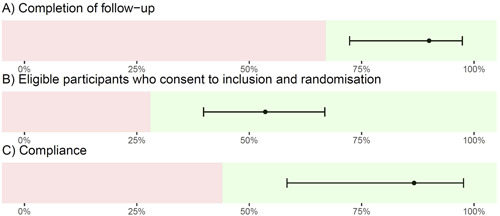

**Key points**

- Specific Non‐suicidal self‐injury (NSSI) treatment recommendations are sparse, so this feasibility trial investigated the internet‐based therapy (ERITA) within Child and Adolescent's Mental Health Services in Denmark as a less resource demanding treatment option as add on to treatment as usual (TAU) in NSSI engaging youth.
- All feasibility outcomes were met: the proportion of randomized eligible patients (54%), the proportion of participants completing the experimental intervention (87%), and the proportion participating in follow‐up interviews (90%).
- Only a limited number of technical issues related to login obstacles occurred during the online provision of the ERITA therapy.

## BACKGROUND

Non‐suicidal self‐injury is defined as *“the deliberate self‐inflicted destruction of body tissue without suicidal intent and for purposes not socially sanctioned”* (International society for the study of self‐injury, 2007). Non‐suicidal self‐injury is increasingly common among vulnerable adolescents and has become a public health challenge of great concern (Jacobson & Gould, 2007). The overall estimated prevalence of NSSI among adolescents is 17% (Muehlenkamp et al., 2012; Swannell et al., 2014), while the prevalence in young psychiatric patients is up to 90% for adolescents with borderline personality disorder displaying emotion regulation difficulties, instability in relationships, or personality disorder traits (Andrewes et al., 2019). Moreover, NSSI is associated with an increased risk of suicidal behavior (Muehlenkamp & Brausch, 2019) and is one of the strongest predictors for suicide attempts (Victor & Klonsky, 2014).

There is evidence in favor of Dialectical Behavior Therapy for Adolescents (DBT‐A) in adolescents with persistent self‐harming behavior and personality disorder traits both in individual trials (McCauley et al., 2018; Mehlum et al., 2014) and meta‐analysis (Kothgassner et al., 2020). A recent meta‐analysis of DBT‐A finds a moderate effect size of self‐harm broadly understood (*g* = −0.44; 95% CI −0.81 to −0.07) (Kothgassner et al., 2021). Evidence of a long‐term effect has also been provided in adolescents with repeat self‐harm (Mehlum et al., 2016, 2019). The lack of NSSI specific outcome reporting, different from a broad definition of self‐harming outcomes, is also the case in dialectical behavior therapy (DBT) trials (Kothgassner et al., 2021). Moreover, DBT‐A is a resource demanding treatment for both the adolescents and services to engage in. Dialectical Behavior Therapy for Adolescents requires four motivational sessions followed by 20 weeks of individual and group sessions, hence out‐patient attendance twice a week in 6 months and, in addition required parent group attendance (Rathus & Miller, 2002); a treatment not readily offered at the early stages of NSSI engagement. There is a lack of large‐scale NSSI intervention trials (Turner et al., 2014). Trials investigating Cognitive Behavior Therapy (CBT), even in brief supposedly low cost intervention programs, are either not superior to standard treatment, are limited in sample sizes, or remain un‐replicated, also without specific NSSI reported measures (Slee et al., 2008; Tyrer et al., 2003; Witt et al., 2021). Specifically, a 12‐week, manualized CBT program for anxiety and depression in youth have been further developed and pilot tested in self‐harming adolescents and referred to as the ‘cutting down program’ (CDP) (Taylor et al., 2011). However, no difference between CDP and TAU was found on self‐harming measures including NSSI in a pilot study (Taylor et al., 2011). More recently, a randomised, clinical trial also investigating CDP compared to TAU (*n* = 74) still found no difference between the group interventions on past month NSSI episodes, however, found that CDP had a faster impact on the self‐injurious behaviour (Kaess et al., 2020). The STAR consortium behind this cognitive intervention has now taken on the task of investigating I‐CDP as an online therapeutic intervention (*Star‐Projekt*). The Swedish and Danish feasibility studies of the internet‐based intervention ERITA (J Bjureberg et al., 2018; Morthorst et al., 2021; Simonsson et al., 2021) plus the completed Swedish randomised, controlled trial (Bjureberg, J. 2022 in review) suggest that ERITA is worthy of further studies.

Recent systematic reviews of e‐mental health and digital interventions for self‐harming behavior, including DBT‐informed apps, present inconsistent findings without enough evidence to currently recommend digital treatment for NSSI (Arshad et al., 2020; Stefanopoulou et al., 2020). This inconsistency is mainly due to methodological limitations in risk of bias among trials in self‐harming populations (Arshad et al., 2020; Stefanopoulou et al., 2020). However, these reviews do conclude that digital interventions are safe and acceptable warranting further investigation also in adolescents with potential barriers towards treatment (Arshad et al., 2020; Stefanopoulou et al., 2020).

To meet the need for short‐term, effective, and easily accessible treatment, an individual, therapist‐supported, Internet based therapy for adolescents *Emotion Regulation Individual Therapy for Adolescents* (ERITA) was developed (Bjureberg et al., 2017). ERITA is a youth‐adapted version of Emotion Regulation Group Therapy (Sahlin et al., 2017) based on elements from DBT, emotion focused therapy, and acceptance and commitment therapy (ACT), which addresses regulation of emotions through skills training. Furthermore, a parent program is included focusing on emotional awareness and how to support their adolescent. ERITA has shown promise in uncontrolled, open trial designs (Bjureberg et al., 2017, 2018; Simonsson et al., 2021), but has yet to be tested in randomised clinical trials.

Internet‐based interventions have not previously been offered in Child and Adolescent Mental Health Services (CAMHS) in Denmark. Before embarking on a larger pragmatic randomised trial, we investigate the feasibility of such a trial. Recent reviews specifically investigating NSSI populations, speculate that digital interventions have the greatest news value in the beginning and then lose ground, where young people lose interest and do not complete the therapeutic intervention (Arshad et al., 2020; Stefanopoulou et al., 2020), so we found it relevant to investigate if internet‐based therapy was of interest to the families referred to CAMHS and whether adherence to treatment was satisfactory before initiating a costly, large‐scale trial.

The TEENS feasibility trial was designed to assess the feasibility of Internet based ERITA in preparation of a large‐scale trial (Morthorst et al., 2021). We hypothesised that it is feasible to test Internet based ERITA in a clinical trial. Specifically, we expected that at least 40% of the eligible patients would be enrolled in this feasibility trial, that at least 73% would complete six treatment modules or more, and that at least 87% would participate in follow‐up assessment at end of the intervention (Olsen et al., 2021).

## OBJECTIVE

To investigate the feasibility of ERITA as add‐on to TAU compared with TAU alone in 13‐17‐year‐old adolescents engaging in NSSI referred to psychiatric outpatient services.

## METHODS

### Study design

The TEENS feasibility trial is an investigator‐initiated randomised clinical trial in a parallel group design with blinded outcome assessment after 12 weeks intervention. The Regional Committee on Health Research Ethics in the Capital Region waived the need for approval (H‐19042904, on September 4th, 2019). The trial was approved by the Knowledge Centre on Data Protection Compliance in the Capital Region (P‐2020‐113, February 2nd, 2020) and registered at clinicaltrials.gov (identification no. NCT04243603, May 11th, 2020) prior to enrollment of the first participant. The trial protocol was published prior to inclusion of the last participant, and a detailed statistical analysis plan was published before any analyses were carried out (Morthorst et al., 2021; Olsen et al., 2021). Data will be publicly stored at The Danish National Archives (DNA) after publication of the trial results (*The Danish National Archives—Rigsarkivet ‐ www.sa.dk, n.d.*). Deidentified individual participant data (including data dictionaries) can be obtained after publication of the trial results at DNA.

Participants were enrolled from CAMHS in the Capital Region of Denmark. Child and Adolescent Mental Health Services provides health care, free of charge based on public taxation, including in‐ and outpatient mental health treatment; referral from the primary health care sector is the norm. The recruitment period was May to October 2020 while the entire trial period, including follow‐up assessments, took place from May 2020 to January 2021.

### Participants

The participants were adolescent outpatients with any psychiatric disorder, 13–17 years of age, who had engaged in at least five NSSI‐episodes during the last year and at least one episode during the past month, assessed by Deliberate Self‐harm Inventory‐Youth (DSHI‐Y) (Gratz, 2001). An additional inclusion criterion was informed consent from legal guardians or caregivers as well as commitment to participate by one parent in the parent part of the program and age‐appropriate literacy assessed by the self‐injury team. There were no exclusion criteria besides imminent suicidal risk requiring in‐patient care.

### Enrolment procedure

A screening procedure for NSSI was implemented in the CAMHS outpatients' services. If NSSI was present, a managing clinician briefly introduced eligible patients and their families of the opportunity to participate in the TEENS feasibility trial. Oral consent was obtained from the family to disclose contact information to the self‐injury team, who then screened for eligibility by calling the families and further introduced the trial design in more detail. Information was provided orally to all patients and legal guardians and in writing also to all eligible persons aged 15 years and above. All members of the self‐injury team are experts in the field of NSSI and experienced in child and adolescent psychiatry including in family consultations and management of mental health vulnerability in youth. Interested families were invited to an online baseline interview. Due to the Covid‐19 situation and because the experimental intervention was Internet based, all dialogue was held by telephone or online. Hereafter, eligible families (legal guardians and youth from 15 years of age) provided written informed consent and completed the electronic baseline questionnaires (see below). The randomisation procedure was handled centrally by the Copenhagen Trial Unit (www.ctu.dk) using a computer‐generated allocation sequence, ratio 1:1 with a varying block size of 6, 8, and 16. Both the allocation sequence and the block size were concealed from the investigators. Due to the feasibility design with a limited sample, we did not apply any stratification variables for the randomisation in this trial. The randomisation was carried out after the baseline interview, and participants allocated to the experimental group were introduced to the electronic platform of the experimental intervention.

To prevent or minimise missing data, the participants were directed to fill out electronic research questionnaires before being able to access the treatment modules of the online ERITA platform. Text message reminders were sent automatically to participants who did not complete their weekly assessment. Prior to the follow‐up interviews, unblinded personnel accessed the research database to make sure that the electronic post‐assessment questionnaire was filled out. If not, a direct link to the assessment was sent to the participants' cell phone.

### Treatment groups (Interventions)

*Experimental intervention –* The Internet based ERITA intervention as an add‐on to TAU consisting of an introduction plus 11 modules (one each week for 12 weeks) of manualized Internet based therapy. The experimental intervention includes psychoeducation, and skills training in impulse control, emotion awareness, acceptance, and validation. The format of the program consists of theoretical texts, animated films, audios, illustrations, case examples of emotion regulation theory, and interactive exercises, as well as a message function for online communication with the assigned ERITA therapist.

ERITA also provides six separate modules for the parents. The parent modules provide psychoeducation on NSSI and other destructive behaviors, NSSI functions, emotional awareness, effective communication skills (e.g., validation), and strategies to cope with the child's negative emotions in a constructive manner. For further details of the ERITA therapy and program, please see (Bjureberg et al., 2018).

The participants and the parents are assigned the same therapist throughout the ERITA program. The online communication with the therapist takes place through an asynchronous message function, and is meant for clarification of the theory, motivation to comply with the program, validation of the efforts provided by the participant, and assistance in testing the new skills. The therapist reviews the participant's responses and provides written feedback to the participant several times a week. The adolescents are asked to complete one chapter every week while the parents are asked to complete a chapter every second week and review the content of the youth chapter every week. A mobile app is available to complement the Internet based treatment. The app provides the participant with the opportunity to report on both self‐destructive impulses and behaviors daily. The mobile app includes reminders of homework assignments and several built‐in programs reminding of the skills acquired in the Internet based program, for example, impulse control, and emotion regulation skills.

*Control intervention –* TAU was provided by CAMHS as a variety of outpatient treatments in both groups. Treatment as usual consists of clinical assessment and treatment for the participants' primary psychiatric condition such as, for example, obsessive‐compulsive disorder, eating disorders, psychoses, or affective disorders. The treatment provided covers pharmacological treatment, Family‐Based Treatment, cognitive behavioral therapy (CBT), DBT, supportive counselling, and/or psychoeducation as needed for the individual patient according to evidence‐based recommendations and guidelines applied in CAMHS (Witt et al., 2021).

### Outcomes

#### Feasibility outcomes

The feasibility outcomes were (1) the proportion participants who completed follow‐up assessment; (2) the proportion of eligible patients who were included in the trial; and (3) the proportion of participants in the experimental intervention group who completed at least six of the ERITA modules. Completion of follow‐up assessment was defined as completing the clinical outcome of DSHI‐Y at end of intervention by telephone interview or electronic questionnaire. Eligibility was defined as those who fulfilled the inclusion criteria and proceeded to randomisation. Completion of ERITA was defined as the adolescent participants completing at least six of 11 ERITA modules as registered by session completion automatically saved on the ERITA platform by the participant once they have gone through a chapter. The rationale for this was that from module one to six, new theory and insight into self‐injures behavior and emotion regulation are introduced. Although concepts such as validation and valued direction are introduced in the second half of the treatment, these latter modules mostly serve as repetition and consolidation of what has already been taught (Bjureberg et al., 2017).

For further feasibility outcome definitions as well as considerations of sample size calculations and power estimations, please see published design paper and statistical analysis plan (Morthorst et al., 2021; Olsen et al., 2021).

#### Explorative clinical outcomes

The *primary explorative clinical outcome* was the number of NSSI episodes within the last 4 weeks at post‐intervention assessment measured by DSHI‐Y by telephone or electronic questionnaire.

The *secondary explorative outcomes* were quality of life assessed with Kidscreen‐10 (Ravens‐Sieberer et al., 2010), symptoms of depression, anxiety, and stress, assessed with Depression, Anxiety, and Stress Scale (Henry & Crawford, 2005), the proportion of participants with any NSSI during the past four weeks, and days absent from school.

*Further exploratory clinical outcomes* were difficulties in emotion regulation assessed with Difficulties in Emotion Regulation Scale (DERS‐16) (Bjureberg et al., 2016), number of indirect self‐destructive behaviors for example, alcohol consumption or drug use assessed with Borderline Symptom List (BSL‐supplement) (Bohus et al., 2009), parents' ability to cope with children's negative emotions rated by adolescents assessed with The Coping with Children's Negative Emotions Scale (CCNES‐APP) (Fabes et al., 2002), and finally adverse events of therapy assessed with Negative Effects Questionnaire (NEQ) (Rozental et al., 2016).

#### Statistical analysis

We published a protocol article including a description of the sample size calculation (Morthorst et al., 2021) and we followed our pre‐published statistical analysis plan (Olsen et al., 2021). Baseline characteristics were analysed using descriptive statistics. The feasibility outcomes were investigated using 1‐sample proportions test with continuity correction, where the lower confidence limit (LCL) of the feasibility outcome should be equal to or above the defined thresholds (Olsen et al., 2021). Dichotomous outcomes were investigated using logistic regression, while count outcomes were analysed using Mann‐Whitney U and presented using the point estimate and the 95% Hodghes‐Lehman derived CIs. The continuous outcomes did not fulfil the assumptions for linear regression, and were analysed using the methodology for the count outcomes (Olsen et al., 2021). A general recommendation or rule of thumb is not to include less than 12 participants in each group in pilot studies (Julious, 2005). Due to lack of previous randomized, clinical trials we pragmatically chose to include 15 participants in each group to provide some robustness for the sample size calculation and power estimation of a future randomised clinical trial also taking the risk of missing data of the explorative outcomes in this trial into account (Julious, 2005; Olsen et al., 2021). For further details, please see the statistical analysis plan (Olsen et al., 2021).

## RESULTS

**FIGURE 1:**
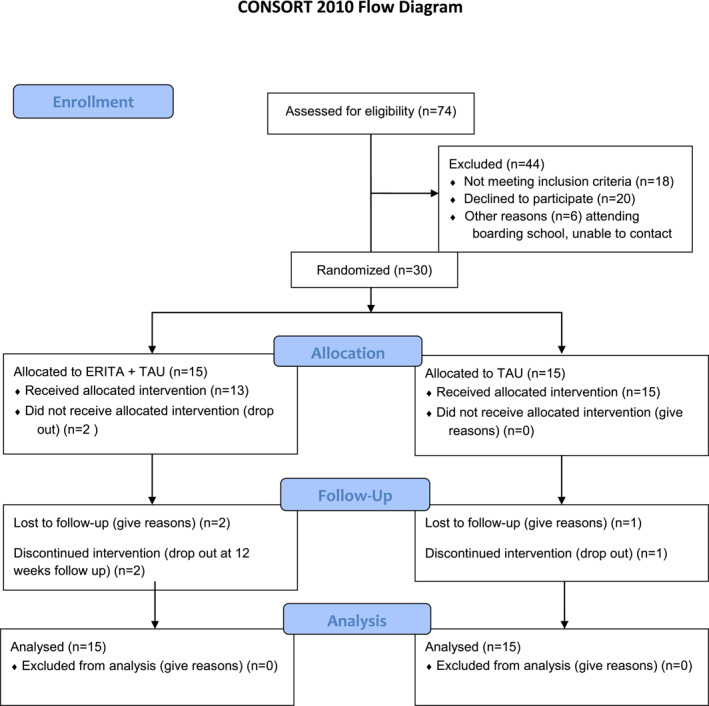
Flowchart

Between May and October 2020, 74 patients were assessed for eligibility, an assessment leading to 56 patients fulfilling the inclusion criteria of which 30 were included and randomised resulting in 15 participants in each intervention group (Figure 1). The majority identified themselves as of female gender (96.7%) and the mean age among all participants was 15 years (standard deviations (SD), 1.35). Most participants attended middle‐school and the number of participants living with married or divorced parents was equally randomised between the two groups. Affective disorders and autism spectrum disorders were the most prevalent diagnoses among the participants and the mean number of NSSI episodes during the past month at baseline was 13.8 (SD 13.9). In general, no apparent baseline group differences were identified (Table 1; Table S1). The median deviation from the planned 12‐week follow‐up was 2 days (interquartile range, 1–3).

**TABLE 1:** Baseline demographics and clinical characteristics

Characteristic	ERITA^a^ (*n* = 15)	TAU^b^ (*n* = 15)	Overall
Age, mean (SD)	15.20 (1.32)	14.87 (1.41)	15.03 (1.35)
Gender (%)			
Female	15 (100.0)	14 (93.3)	29 (96.7)
Transgender	0 (0.0)	1 (6.7)	1 (3.3)
Nationality (%)			
Danish	14 (93.3)	15 (100.0)	29 (96.7)
European	1 (6.7)	0 (0.0)	1 (3.3)
School (%)			
Boarding school^c^	1 (6.7)	1 (6.7)	2 (6.7)
High school	2 (13.3)	2 (13.3)	4 (13.3)
Middle school	10 (66.7)	9 (60.0)	19 (63.3)
No school	1 (6.7)	1 (6.7)	2 (6.7)
Other^d^	1 (6.7)	2 (13.3)	3 (10.0)
Psychiatric diagnosis (ICD‐10, A‐diagnosis) (%)			
F30‐39 (Affective disorders)	4 (26.7)	4 (26.7)	8 (26.7)
F40‐49 (Anxiety disorders)	1 (6.7)	1 (6.7)	2 (6.7)
F50‐59 (Eating disorders)	2 (13.3)	0 (0.0)	2 (6.7)
F60‐69 (Personality disorders)	0 (0.0)	1 (6.7)	1 (3.3)
F80‐89 (Developmental and autism spectrum disorders)	3 (20.0)	5 (33.3)	8 (26.7)
F90‐98 (Behavioral disorders)	3 (20.0)	3 (20.0)	6 (20.0)
Z032 (Medical observation and assessment for mental disorders)	2 (13.3)	1 (6.7)	3 (10.0)
Comorbid diagnoses (ICD‐10, B‐diagnosis) (%)			
F30‐39 (Affective disorders)	2 (13.3)	1 (6.7)	3 (10.0)
F40‐49 (Anxiety disorders)	6 (40.0)	5 (33.3)	11 (36.7)
F50‐59 (Eating disorders)	1 (6.7)	0 (0.0)	1 (3.3)
F60‐69 (Personality disorders)	4 (26.7)	3 (20.0)	7 (23.3)
F70‐79 (Mental retardation)	0 (0.0)	2 (13.3)	2 (6.7)
F80‐80 (Developmental and autism spectrum disorders)	2 (13.3)	0 (0.0)	2 (6.7)
F90‐98 (Behavioral disorders)	2 (13.3)	2 (13.3)	4 (13.3)
X60.1 (Suicide attempt, non‐opioid analgesics)	1 (6.7)	0 (0.0)	1 (3.3)
X78.2 (Suicide attemps, sharp object)	3 (20.0)	1 (6.7)	4 (13.3)
Z032 (Medical observation and assessment for mental disorders)	1 (6.7)	0 (0.0)	1 (3.3)
Z60 (Social environmental problems)	1 (6.7)	0 (0.0)	1 (3.3)
Z61 (Traumatic childhood events)	1 (6.7)	2 (13.3)	3 (10.0)
Z62 (Problems related to upbringing)	0 (0.0)	1 (6.7)	1 (3.3)
Z63 (Problems related to relatives)	2 (13.3)	1 (6.7)	3 (10.0)
Z81 (Anamnestic psychiatric disorder)	1 (6.7)	1 (6.7)	2 (6.7)
Z91 (Other risk factors)	0 (0.0)	1 (6.7)	1 (3.3)
Parental status (%)			
Divorced	7 (46.7)	7 (46.6)	14 (46.7)
Married	8 (53.3)	7 (46.7	15 (50.0)
Other	0 (0.0)	1 (6.7)	1 (3.3)

^a^ ERITA, Emotion Regulation Individual Therapy for Adolescents, the internet‐based intervention + TAU.

^b^ TAU consists of varies outpatient treatment offers provided in Child and Adolescents Mental Health Services in the Capital Region Denmark.

^c^ Attendance of boarding school was possible during the internet based ERITA course if wished for by the participating adolescent. Boarding school was not an exclusion criterion, but this was perceived as a barrier for selected participants who therefore declined to participate.

^d^ Day college as preparation for subsequent schooling.

### Feasibility outcomes

**FIGURE 2:**
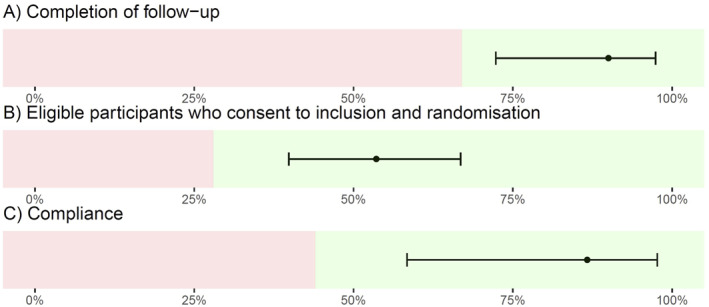
Feasibility outcomes.^a^ *Note*: ^a^ The feasibility outcomes were investigated using 1‐sample proportions test with continuity correction, with the lower confidence as limit of the feasibility outcomes.

The proportion of participants who completed follow‐up was 90% (95% CI, 72%–97%) (Table 2), two participants in ERITA and one in TAU were lost to follow‐up (Figure 2). Of the 15 participants allocated to the ERITA group, two dropped out: one even before initiating the program and one after completing the introduction and the first module. Two participants did not complete the program from module six to 11, while one skipped module seven but then continued and completed the rest of the modules.

**TABLE 2:** Feasibility outcomes

Outcome	Requirement^a^	Fraction	LCL	UCL
Completion of follow‐up^b^	68%	90%	72%	97%
Eligible participants who consent to inclusion and randomization	27%	54%	40%	67%
Compliance^c^	45%	87%	58%	98%

Abbreviations: LCL, lower confidence limit; UCL, upper confidence limit.

^a^ The LCL must ≥ requirement.

^b^ Participating in at least one follow‐up assessment.

^c^ Completing at least six out of eleven ERITA‐modules.

The proportion of eligible individuals who consented to participation and randomisation was 54% (95% CI, 40%–67%), and the proportion of compliance with at least six of 11 ERITA modules was 87% (95% CI, 58%–98%).

### Explorative clinical outcomes

No statistically significant difference was identified between groups on the primary explorative clinical outcome of NSSI episodes at follow‐up (point estimate: 1; 95% CI, −1 to 4, *p = 0*.*20*). Similarly, no group difference was identified for most of the other explorative clinical outcomes (Table 3). However, the experimental intervention group did show fewer risk‐related events assessed by BSL‐supplement (point estimate: 1; 95% CI, 0–3, *p =* 0.02). We observed no difference between the two groups regarding negative event measures assessed by NEQ (point estimate: 0; 95% CI, −5 to 3, *p* = 0.97) (Table 3).

**TABLE 3:** Explorative clinical outcomes^a^

Outcomes	ERITA^b^ (*n* = 15)	TAU^c^ (*n* = 15)	Estimate (95% CI)	*p*‐value
DSHI‐Y, mean (SD) [*n*]				
Baseline	13.20 (11.60) [15]	12.73 (16.07) [15]		
Follow‐up	3.46 (6.62) [13]	6.71 (9.63) [14]	1 (−1 to 4)	0.2007
KIDSCREEN‐10 T‐values, mean (SD) [*n*]				
Baseline	33.11 (4.79) [15]	35.61 (5.82) [15]		
Follow‐up	33.01 (5.78) [11]	35.13 (6.69) [12]	1.91 (−3.82–7.37)	0.423
DASS‐21, mean (SD) [*n*]				
Depression baseline	12.60 (4.60) [15]	12.20 (4.97) [15]		
Depression follow‐up	10.91 (5.52) [11]	11.58 (6.50) [12]	0.73 (−5 to 7)	0.9018
Stress baseline	10.53 (2.80) [15]	11.13 (3.87) [15]		
Stress follow‐up	11.09 (4.61) [11]	11.00 (4.81) [12]	0 (−5 to 4)	0.9017
Anxiety baseline	8.33 (4.19) [15]	7.53 (4.81) [15]		
Anxiety follow‐up	8.27 (4.90) [11]	5.67 (4.38) [12]	−2 (−7 to 2)	0.291
Self‐Injury^d^, N (%) [*n*]				
OR	9 (69.2) [13]	11 (78.6) [14]	0.61 (0.10–3.50)	0.582
RR^e^	9 (69.2) [13]	11 (78.6) [14]	0.88 (0.48–1.28)	
BSL‐supplement				
Baseline	4.73 (3.75) [15]	4.07 (3.31) [15]		
Follow‐up	1.00 (2.00) [11]	2.92 (3.18) [12]	1 (0–3)	0.0235
CCNES‐APP				
Distress reaction (follow‐up)	2.29 (0.98) [11]	2.29 (0.95) [12]	0 (−0.89 to 0.89)	0.9754
Punitive reactions (follow‐up)	2.16 (1.02) [11]	1.90 (1.16) [12]	−0.33 (−1.22–0.33)	0.3083
Expressive encouragement (follow‐up)	3.36 (0.94) [11]	4.06 (1.53) [12]	0.67 (−0.56–2)	0.1842
Emotion‐focused reactions (follow‐up)	4.10 (1.36) [11]	4.29 (1.64) [12]	0.22 (−1 to 1.67)	0.6657
Problem‐focused reactions (follow‐up)	4.27 (1.53) [11]	4.82 (1.44) [12]	0.44 (−0.67–2)	0.3884
Minimization reactions (follow‐up)	2.98 (1.29) [11]	2.69 (1.00) [12]	−0.26 (−1.56–0.78)	0.6888
NEQ				
NEQ Freq. negative effects	7.00 (4.60) [11]	6.64 (5.26) [11]	0 (−5 to 3)	0.9737
NEQ Freq. negative effects from treatment	3.82 (3.60) [11]	2.55 (3.05) [11]		
NEQ Freq. negative effects from other circumstances	3.18 (2.89) [11]	4.09 (3.14) [11]		
NEQ Freq. Fac. 1 (Symptoms) from treatment	1.55 (1.69) [11]	0.55 (1.04) [11]		
NEQ Freq. Fac. 2 (Quality) from treatment	1.55 (1.69) [11]	1.36 (1.91) [11]		
NEQ Freq. Fac. 3 (Dependency) from treatment	0.00 (0.00) [11]	0.09 (0.30) [11]		
NEQ Freq. Fac. 4 (Stigma) from treatment	0.55 (0.82) [11]	0.09 (0.30) [11]		
NEQ Freq. Fac. 5 (Hopelessness) from treatment	0.18 (0.40) [11]	0.45 (0.69) [11]		
NEQ Neg. impact from treatment	11.45 (12.82) [11]	7.00 (9.21) [11]		
NEQ Neg. impact Fac. 1 (Symptoms) from treatment	4.09 (5.61) [11]	1.45 (2.73) [11]		
NEQ Neg. impact Fac. 2 (Quality) from treatment	4.82 (5.90) [11]	3.27 (5.22) [11]		
NEQ Neg. impact Fac. 3 (Dependency) from treatment	0.00 (0.00) [11]	0.27 (0.90) [11]		
NEQ Neg. impact Fac. 4 (Stigma) from treatment	2.00 (3.19) [11]	0.27 (0.90) [11]		
NEQ Neg. impact Fac. 5 (Hopelessness) from treatment	0.55 (1.29) [11]	1.73 (3.07) [11]		

Abbreviation: CCNES‐APP, Coping with Children's Negative Emotions Scales ‐ Adolescents' parent perception.

^a^ Due to lack of normal distribution non‐parametric tests were applied.

^b^ TAU consists of varies outpatient treatment offers provided in Child and Adolescents Mental Health Services in the Capital Region Denmark.

^c^ ERITA, Emotion Regulation Individual Therapy for Adolescents, the internet‐based intervention.

^d^ Self‐injury dichotomized as secondary, explorative outcome at follow‐up.

^e^ CIs (CI) of risk ratio (RR) are Unconditional Maximum Likelihood Estimation & normal approximation (Wald), while the CI for RR will be obtained using marginal effects.

## DISCUSSION

This randomised clinical feasibility trial included outpatient adolescents in mental health care who engaged in NSSI. We showed that it was feasible to investigate ERITA as an addition to TAU compared with TAU alone in a randomised trial design. The proportion of participants who completed the follow‐up assessment, the proportion that was included in the trial, and the proportion that followed the ERITA intervention were all above our pre‐set criteria for feasibility.

A 90% completion at follow‐up by interview assessment is considered excellent in trials of psychosocial interventions (Fisher et al., 2019; Witt et al., 2021). The finding that more than half of the eligible patients willingly participated, further vouches for the feasibility of a subsequent large‐scale randomised clinical trial with sufficient power. ERITA has previously been found feasible within adolescent psychiatric services in studies with uncontrolled designs (Bjureberg et al., 2017, 2018). A strength of the present feasibility trial is the randomised design, which the participants seemingly accepted. This bodes well for trial participation in a subsequent randomised clinical trial in low risk of selection bias. One should note that the lower limit of the 95% CI on completion drops down to 72%. Such low figures would endanger the conclusiveness of a randomised clinical trial. Accordingly, additional efforts to increase follow up should be considered, although evidence for strategies to improve retention in randomised trials is weak (Gillies & Aceves‐Martins, 2021).

As evident in Table 1, a large part of our sample has a primary diagnosis of developmental and autism spectrum disorders. Though NSSI is associated with several psychiatric disorders (Nock et al., 2006; Segers & Rawana, 2014), it is possible that individuals with autism spectrum disorder engaging in NSSI (Moses, 2018) may find the internet‐based design especially appealing due to the structured learning approach and a therapeutic communication form based on writing as is the case with this internet‐based treatment of ERITA. This aspect calls for further investigation.

Recent reviews showed that digital interventions including mobile apps and Internet based interventions are safe and acceptable also for adolescents (Arshad et al., 2020; Stefanopoulou et al., 2020). As for the findings of the safety measure of NEQ, we did not observe any indications of group differences regarding negative events, but our sample size is limited. The pronounced variation in NEQ data combined with the limited sample, implied numeric noise. In a future large‐scale trial, we may have sufficient power to show differences for subgroup item calculations of negative impacts.

An impact of ERITA on risk behaviors, for example, alcohol consumption or illegal drug use assessed by the BSL‐supplement tool has previously been found in a Swedish, uncontrolled, pilot study (Bjureberg et al., 2018). However, this finding may imply a risk of a type 1 error, due to the limited power and limited testing. With a population of 30 participants the current findings are only exploratory and not valid as efficacy measures. The finding regarding the BSL‐supplement reduction could lead to a consideration of making this a secondary instead of an explorative outcome in a large‐scale trial.

During the present feasibility trial, in preparation of a large‐scale trial, we learned that the continuous outcomes assessed were not normally distributed which means that we had to apply non‐parametric tests. Most of the analyses in this trial were non‐parametric tests; however, in a large‐scale trial, some outcomes might conform to normal distribution.

We also learned that the outcome of sick days within the last 4 weeks was not easily handled. The outcome was chosen as a ready‐at‐hand, countable proxy for the level of functioning among the participants, but we have learned that this question was difficult to understand and answer for the participants. We kept getting questions on which kind of sick leave this referred to, mentally or physically and since most participants were sent home during the COVID‐19 lock down this item was troublesome and to some extent invalid. Also, many of the participants had individual schemes of school attendance to follow other therapeutic treatment options in TAU, making comparison with ordinary school attendance less straight forward. Due to missing data and the abovementioned limitations, we opted from presenting this outcome and have respecified the wording of this item in the future large‐scale trial distinguishing the expected number of school days and the actual number of attendances for each adolescent.

During the feasibility trial we also learned that the need for technical support for the participants to operate on the ERITA platform was limited with only minor technical obstacles, mostly related to access to the intervention platform. Despite extensive pre‐testing, we learned that electronic data collection of CCNES‐APP at baseline and of emotion regulation by DERS‐16 at follow‐up was uninstalled; a trial error that will be corrected in a subsequent large‐scale trial.

The TEENS feasibility trial was limited by not being able to blind the participants as well as the ERITA therapists providing the experimental intervention; a limitation that is inherent in psychosocial intervention trials (Juul et al., 2021). However, to overcome potential bias we performed blinded outcome assessment as well as blinded statistical analyses by two independent statisticians including a consensus report and two abstracts for the steering committee to agree upon before performing the unblinding; all intentions in preparation for a large‐scale trial potentially at several sites with additional teams providing the ERITA intervention in other Danish regions to further improve the external validity. The findings of a full‐scale Swedish trial are currently under review (Bjureberg, J. et al., in review 2022). If evidence is provided for more benefits than harms of the ERITA therapy based on future large‐scale trials, this intervention could be considered in primary sectors and social services as up‐stream prevention in the early stages of NSSI.

## CONCLUSIONS

The TEENS feasibility trial showed feasibility of the research and treatment protocols. All the feasibility outcomes were met; therefore, it is legitimate to conclude that a large‐scale trial is feasible and predict that self‐injuring adolescents will engage in internet‐based ERITA. In a large‐scale randomised clinical trial, we wish to assess if internet‐based ERITA intervention is a valuable addition to TAU for adolescents engaging in NSSI.

## AUTHOR CONTRIBUTIONS

**Britt Morthorst**: Conceptualization, Data curation, Formal analysis, Funding acquisition, Investigation, Methodology, Project administration, Resources, Software, Validation, Visualization, Writing – original draft, Writing – review & editing. **Markus Olsen**: Formal analysis, Methodology, Software, Supervision, Validation, Visualization, Writing – review & editing. **Janus Jakobsen**: Conceptualization, Formal analysis, Methodology, Resources, Software, Supervision, Validation, Visualization, Writing – review & editing. **Jane Lindschou**: Conceptualization, Formal analysis, Funding acquisition, Methodology, Resources, Software, Supervision, Validation, Visualization, Writing – review & editing. **Christian Gluud**: Conceptualization, Formal analysis, Funding acquisition, Methodology, Resources, Software, Supervision, Validation, Visualization, Writing – review & editing. **Michella Heinrichsen**: Conceptualization, Data curation, Investigation, Methodology, Resources, Software, Validation, Visualization, Writing – review & editing. **Bo Moehl**: Conceptualization, Funding acquisition, Investigation, Resources, Supervision, Validation, Writing – review & editing. **Lotte Rubæk**: Conceptualization, Data curation, Funding acquisition, Investigation, Methodology, Project administration, Resources, Software, Validation, Writing – review & editing. **Johan Bjureberg**: Conceptualization, Data curation, Funding acquisition, Investigation, Methodology, Resources, Software, Supervision, Validation, Visualization, Writing – review & editing. **Anne Katrine Pagsberg**: Conceptualization, Funding acquisition, Investigation, Methodology, Project administration, Resources, Supervision, Validation, Writing – review & editing.

## CONFLICTS OF INTEREST

The authors have declared that they have no competing or potential conflicts of interest.

## ETHICAL CONSIDERATIONS

The Regional Committee on Health Research Ethics in the Capital Region Denmark waived the need for approval (H‐19042904, on September 4th, 2019) while the feasibility trial was approved by the Knowledge Centre on Data Protection Compliance in the Capital Region Denmark (P‐2020‐113, February 2nd, 2020). Following oral and written trial information electronic consent was obtained from all eligible families (legal guardians and youth from 15 years of age) wishing to participate. Age‐appropriate trial information was developed and distributed to adolescents from 15 years and older. For participants allocated to the experimental group receiving ERITA, parents had access to a pdf. with the theoretical part of the adolescent ERITA content to support the young participant, however, the parents had no access to the personal online dialogue between the adolescent and the therapist, which was confidential.

## TRIAL REGISTRATION

ClinicalTrials.gov, NCT04243603. Registered 28 January 2020, https://clinicaltrials.gov/ct2/show/NCT04243603.

## Supporting information

Supplementary Material 1

## Data Availability

Data will be publicly stored at The Danish National Archives (DNA) after publication of the trial results (The Danish National Archives ‐ Rigsarkivet ‐ Www.Sa.Dk, n.d.). Deidentified individual participant data (including data dictionaries) can be obtained after publication of the trial results at DNA.
