# Supplementary material for: Internet based intervention (Emotion Regulation Individual Therapy for Adolescents) as add‐on to treatment as usual versus treatment as usual for non‐suicidal self‐injury in adolescent outpatients: The TEENS randomised feasibility trial
Source: JCPP Adv. 2022 Dec 3;2(4):e12115. doi: 10.1002/jcv2.12115 (PMC10242949; doi:10.1002/jcv2.12115)
Supplement: Supplementary file 1 — Supplementary Material 1 [file JCV2-2-e12115-s001.docx]

**Supporting Information**

| Table S1. Number and type of psychiatric treatments provided in treatment as usual (TAU) in both groups (n=30) | | | |
| --- | --- | --- | --- |
| Types of treatment | **ERITA^a^**  (n=15) | **TAU^b^**  (n=15) | **Total** |
| Visit to emergency department | 2 | 2 | 4 |
| Admission; somatic and psychiatric | 2 | 0 | 2 |
| Psychiatric/diagnostic assessment | 4 | 5 | 9 |
| Medication consultations | 7 | 6 | 13 |
| (Family) consultations, therapy, and status meetings | 21 | 20 | 41 |
| Suicide risk assessments and consultations | 4 | 4 | 8 |
| Individual psychotherapy | 12 | 10 | 22 |
| Group therapy | 3 | 2 | 5 |
| Physiotherapy | 1 | 0 | 1 |
| Parent group therapy | 1 | 0 | 1 |

^a^Treatment as usual (TAU) consists of varies outpatient treatment offers provided in Child and Adolescents Mental Health Services in the Capital Region Denmark.

^b^ ERITA, Emotion Regulation Individual Therapy for Adolescents, the internet-based intervention + TAU.
